# Aqueous extract of *Terminalia arjuna *prevents carbon tetrachloride induced hepatic and renal disorders

**DOI:** 10.1186/1472-6882-6-33

**Published:** 2006-09-30

**Authors:** Prasenjit Manna, Mahua Sinha, Parames C Sil

**Affiliations:** 1 Department of Chemistry, Bose Institute, 93/1, Acharya Prafulla Chandra Road, Kolkata-700009, India

## Abstract

**Background:**

Carbon tetrachloride (CCl_4_) is a well-known hepatotoxin and exposure to this chemical is known to induce oxidative stress and causes liver injury by the formation of free radicals. Acute and chronic renal damage are also very common pathophysiologic disturbances caused by CCl_4_. The present study has been conducted to evaluate the protective role of the aqueous extract of the bark of *Termnalia arjuna *(TA), an important Indian medicinal plant widely used in the preparation of ayurvedic formulations, on CCl_4 _induced oxidative stress and resultant dysfunction in the livers and kidneys of mice.

**Methods:**

Animals were pretreated with the aqueous extract of TA (50 mg/kg body weight) for one week and then challenged with CCl_4 _(1 ml/kg body weight) in liquid paraffin (1:1, v/v) for 2 days. Serum marker enzymes, namely, glutamate pyruvate transaminase (GPT) and alkaline phosphatase (ALP) were estimated in the sera of all study groups. Antioxidant status in both the liver and kidney tissues were estimated by determining the activities of the antioxidative enzymes, superoxide dismutase (SOD), catalase (CAT) and glutathione-S-transferase (GST); as well as by determining the levels of thiobarbutaric acid reactive substances (TBARS) and reduced glutathione (GSH). In addition, free radical scavenging activity of the extract was determined from its DPPH radical quenching ability.

**Results:**

Results showed that CCl_4 _caused a marked rise in serum levels of GPT and ALP. TBARS level was also increased significantly whereas GSH, SOD, CAT and GST levels were decreased in the liver and kidney tissue homogenates of CCl_4 _treated mice. Aqueous extract of TA successfully prevented the alterations of these effects in the experimental animals. Data also showed that the extract possessed strong free radical scavenging activity comparable to that of vitamin C.

**Conclusion:**

Our study demonstrated that the aqueous extract of the bark of TA could protect the liver and kidney tissues against CCl_4_-induced oxidative stress probably by increasing antioxidative defense activities.

## Background

Exposure to various organic compounds including a number of environmental pollutants and drugs can cause cellular damages through metabolic activation of those compounds to highly reactive substances such as reactive oxygen species (ROS). Free radical induced lipid peroxidation is believed to be one of the major causes of cell membrane damage leading to a number of pathological situations [1-3]. Reports from our laboratory and other investigators have established that the industrial solvent, carbon tetrachloride (CCl_4_) is a potent environmental hepatotoxin [4-7]. A number of recent reports clearly demonstrated that in addition to hepatic problems, CCl_4 _also causes disorders in kidneys, lungs, testis and brain as well as in blood by generating free radicals [8-11]. Reports from Perez et al, Ogeturk et al and Churchill et al suggested that exposure to this solvent causes acute and chronic renal injuries [12-14]. In addition, reports on various documented case studies established that CCl_4 _produces renal diseases in humans [15,16]. Extensive evidence demonstrated that ^.^CCl_3 _and ^.^Cl are formed as a result of the metabolic activation of CCl_4_, which in turn, initiate lipid peroxidation process. A known potent antioxidant, vitamin E, could protect CCl_4 _induced liver injury indicating that oxidative stress is responsible for CCl_4 _induced hepatic disorder in this particular model [17,18]. Studies also showed that various herbal extracts could protect organs against CCl_4 _induced oxidative stress by altering the levels of increased lipid peroxidation, and enhancing the decreased activities of antioxidant enzymes, like superoxide dismutase (SOD), catalase (CAT) and glutathione-S-transferase (GST) as well as enhanced the decreased level of the hepatic reduced glutathione (GSH) [19,20]. Knowledge on the protective mechanisms against toxin and drug induced organ-toxicities leads scientists to look for biologically active relevant compounds from herbal plants, which can possess intrinsic antioxidant activity and protect those organs from unwanted oxidative stress. In the modern medicine, plants occupy a significant birth as raw materials for some important drug preparations [21-23]. India is well known for a plethora of medicinal plants. The traditional Indian medicinal plants act as antiradicals and DNA cleavage protectors [24]. These plants have also been considered to protect health, longevity, intelligence, immunosurveillance and body resistance against different infections and diseases. *Tephrosia purpurea *[25], *Silybum marianum *[26], *Picrorhiza kurroa *[27], *Cajanus indicus *[28,29], *Phyllanthus niruri *[30-32], etc. posses hepatoprotective property against different toxins and drugs induced hepatic disorders. *Terminalia arjuna *(TA) is also an important medicinal plant widely used in the preparation of ayurvedic formulations for over three centuries primarily as a cardiac tonic in India [33]. Clinical evaluation of this plant indicates that it can be of benefit in the treatment of coronary artery diseases, heart failure and possibly hypercholesterolemia [34-36]. It has also been found to be antibacterial and antimutagenic [37-39]. However, most of the beneficial works on this plant have been carried out on the alcoholic extract of its bark and very little is known about its role on toxin-induced either hepatic or renal disorders. In this particular study, protective role of aqueous extract of the bark of TA was evaluated against CCl_4_-induced toxicity in the liver and kidney. Firstly, the radical scavenging activity of the extract was determined from its 2,2-diphenyl-1-picryl hydrazyl (DPPH) radical quenching ability and the data were compared to those obtained from a known free radical scavenger, vitamin C. Secondly, the dose- and time-dependent effects of the extract against CCl_4_-induced toxicity were evaluated by measuring the levels of the serum marker enzymes, glutamate pyruvate transaminase (GPT) followed by determining its effect on another serum marker enzyme, alkaline phosphatase (ALP) using optimum dose and time. Finally, hepatic and renal oxidant-antioxidant status was evaluated by measuring the levels of a) antioxidant enzymes SOD, CAT and GST; b) ROS scavenger GSH and c) extent of lipid peroxidation in both the livers as well as the kidneys in mice. In addition, study on the effect of a known antioxidant, vitamin E, was also included against CCl_4 _induced hepatic and renal oxidative stress.

## Methods

### Plant

*Terminalia arjuna *(TA), belonging to the family Combretaceae, has a long history of medicinal uses in India. It is a shade and ornamental tree. The bark of the tree is useful as an anti-ischemic and cardioprotective agent in hypertension and in ischemic heart disease. The bark was collected from local markets.

### Animals

Swiss albino mice (male, body weight 20 ± 2 g) were acclimatized under laboratory condition for a fortnight before starting experiments. They were provided with standard diet and water ad libitum. The animals were divided into four groups, each group having six mice.

### Chemicals

Bradford reagent, bovine serum albumin (BSA), DPPH, and protein estimation kit were purchased from Sigma-Aldrich Chemical Company, (St. Louis, MO) USA. CCl_4_, 1-chloro-2,4-dinitrobenzene (CDNB), 5,5'-dithiobis(2-nitrobenzoic acid) [DTNB, (Ellman's reagent)], disodium hydrogen phosphate (Na_2_HPO_4_), ethylene diamine tetraacetic acid (EDTA), glacial acetic acid, hydrogen peroxide (H_2_O_2_), nicotinamide adenine dinucleotide reduced (NADH), nitro blue tetrazolium (NBT), phenazine methosulphate (PMT), potassium dihydrogen phosphate (KH_2_PO_4_), reduced glutathione (GSH), sodium dihydrogen phosphate (NaH_2_PO_4_), sodium pyrophosphate, trichloro acetic acid (TCA), thiobarbituric acid (TBA), vitamin C, vitamin E were bought from Sisco research laboratory, India.

### Preparation of aqueous extract of TA

The bark of TA was cut into pieces and was homogenized in 50 mM sodium phosphate buffer; pH 7.2, at 4°C and the homogenate was centrifuged at 12,000 g for 30 minutes to get rid of unwanted debris. The supernatant was dialyzed against ice-cold water and centrifuged again under the same condition. The supernatant was collected and lyophilized. The freeze-dried material was weighed, dissolved in the same phosphate buffer and used for this study.

### Determination of radical scavenger activity in cell-free system

#### Quenching of DPPH radical

The radical scavenger activity of the aqueous TA extract was measured spectrophotometrically using the DPPH radical [40]. Aqueous TA extract at various concentrations were added to DPPH in methanol (125 μM, 2 ml) solution. The final volume was adjusted to 4 ml with water. The solution was shaken and incubated at 37°C for 30 minutes in the dark. The decrease in absorbance of DPPH was measured at 517 nm. Percent inhibition was calculated by comparing the absorbance values of the control and the extract. A parallel experiment was carried out under the same conditions in which TA extract was replaced by vitamin C and used as a positive control.

### Determination of dose-dependent effect of the extract

To determine the dose of the TA extract necessary for the maximum hepatic and renal protection, six different groups of mice were separately treated with six different doses of the extract – 1 mg, 5 mg, 10 mg, 25 mg, 50 mg and 100 mg/kg body weight for 7 days prior to CCl_4 _treatment (in liquid paraffin 1:1, v/v) for 2 days at a dose of 1 ml/kg body weight. Twenty-four hours after the final dose of CCl_4 _administration, all mice were sacrificed. The GPT levels were measured from the blood sera of all the experimental animals.

### Determination of time-dependent effect of the extract

To determine the time needed for the TA extract to exhibit maximum hepatic and renal protection, six different groups of mice were separately treated with the extract at a dose of 50 mg/kg body weight for 1, 3, 5, 7 and 10 days prior to CCl_4 _(1 ml/kg body weight) intoxication. Twenty-four hours after the final dose of CCl_4 _administration, all experimental mice were sacrificed, blood samples were collected and the GPT levels were measured.

### Pre-treatment with the aqueous extract of TA

The pretreatment group was divided into three sub-groups each consisted of six mice. The first group served as normal control. The second group received CCl_4 _orally (1 ml/kg body weight) for 2 days and treated as toxin control. The third group received the aqueous TA extract for 7 days (50 mg/kg body weight) orally followed by CCl_4 _treatment at a dose of 1 ml/kg body weight for 2 days. Mice were sacrificed after 24 hours of the final dose of CCl_4 _administration and blood, livers and kidneys were collected. For the positive control vitamin E (200 mg/kg body weight) was administered orally to a group of 6 mice for 7 days followed by CCl_4 _administration as described earlier.

### Preparation of liver and kidney homogenates

About 200 mg of liver and kidney tissue were homogenized separately in 10 volume of 100 mM KH_2_PO_4 _buffer containing 1 mM EDTA, pH 7.4 and centrifuged at 12,000 g for 30 minutes at 4°C. The supernatant was collected and used for following experiments as described below. Protein concentration of the supernatant was measured according to the method of Bradford [41] using crystalline BSA as standard.

### Assessment of serum specific markers enzymes

Blood samples collected from puncturing mice hearts were kept overnight for clotting and then centrifuged at 3,000 g for 10 minutes. GPT and ALP levels in all the sample sera were estimated by the methods of Rietman and Frankel [42] and Kind and King [43] respectively.

### Assay of antioxidant enzymes in the liver and kidney homogenates

#### SOD assay

The activity of SOD was measured following the method of Nishikimi [44] and then modified by Kakkar [45]. About 5 μg total protein from each of the liver and kidney homogenates were mixed with sodium pyrophosphate buffer, PMT and NBT. The reaction was started by the addition of NADH. The reaction mixture was incubated at 30°C for 90 seconds and stopped by the addition of 1 ml of glacial acetic acid. The absorbance of the chromogen formed was measured at 560 nm. One unit of SOD activity is defined as the enzyme concentration required for the inhibition of chromogen production by 50% in one minute under the assay conditions.

#### CAT assay

The enzyme CAT catalyzes the conversion of H_2_O_2 _into water. The CAT activity was measured by the method of Bonaventura [46]. About 5 μg protein from the liver and kidney homogenates were mixed with 2.1 ml of 7.5 mM H_2_O_2 _and the reaction was allowed to continue for 10 minutes at 25°C. The disappearance of peroxide was continuously recorded from the absorbance at 240 nm for the specified period of time. One unit of CAT activity is defined as the amount of enzyme necessary for reducing1 μmol of H_2_O_2 _per minute.

#### GST assay

GST catalyzes the conjugation reaction with glutathione in the first step of mercapturic acid synthesis. GST activity was measured by the method of Habig and Jakoby [47]. The reaction mixture contained suitable amount of the enzyme (25 μg of protein in homogenates), KH_2_PO_4 _buffer, EDTA, CDNB and GSH. The reaction was carried out at 37°C and monitored spectrophotometrically by the increase in absorbance of the conjugate of GSH and CDNB at 340 nm. A blank was run in absence of the enzyme. One unit of GST activity is 1 μmol product formation per minute.

### Assay of non-enzymatic antioxidant in the liver and kidney homogenates

#### GSH assay

GSH level was measured by the method of Ellman [48]. The homogenate (720 μl) was double diluted and 5% TCA was added to it to precipitate the protein content of the homogenate. After centrifugation (10,000 g for 5 minutes) the supernatant was taken, DTNB solution (Ellman's reagent) was added to it and the absorbance was measured at 412 nm. A standard graph was drawn using different concentrations of a standard GSH solution (1 mg/ml). With the help of the standard graph, GSH contents in the liver and kidney homogenates of the experimental animals were calculated.

### Estimation of lipid peroxidation products

Degree of lipid peroxidation in the liver and kidney tissue homogenates of all the experimental animals was determined in terms of thiobarbituric acid reactive substances (TBARS) formation [49]. The sample containing1 mg protein was mixed with 1 ml TCA (20%), 2 ml TBA (0.67%) and heated for 1 hour at 100°C. After cooling, the precipitate was removed by centrifugation. The absorbance of the sample was measured at 535 nm using a blank containing all the reagents except the tissue homogenate. As 99% of the TBARS is malondialdehyde (MDA), TBARS concentrations of the samples were calculated using the extinction co-efficient of MDA, which is 1.56 × 10^5 ^M^-1^cm^-1^.

### Statistical analysis

All the values are represented as mean ± S.D. (n = 6). Data on biochemical investigations were analyzed using analysis of variance (ANOVA) and the group means were compared by Duncan's Multiple Range Test (DMRT). A probability of p < 0.05 was considered as significant.

## Results

### Effect of the aqueous TA extract on radical scavenger activity in cell-free system

**Figure 1:**
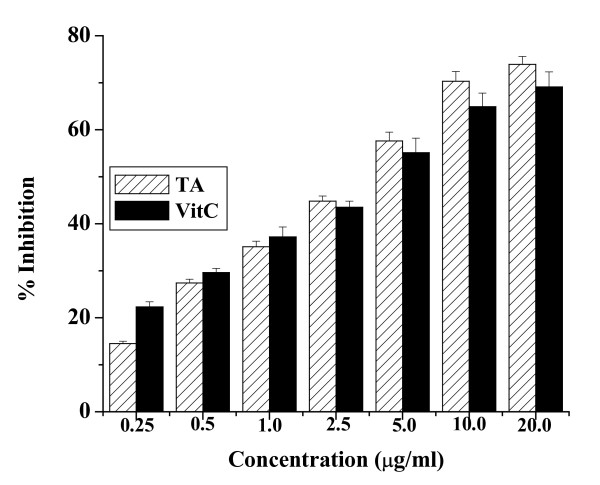
DPPH radical scavenging activity of aqueous TA extract in cell-free system. The curve is obtained by plotting various concentrations of the extract (μg/ml) against percent inhibition of DPPH radical. Vitamin C (VitC) has been used as a known DPPH radical scavenger.

Figure 1 shows the radical scavenging effect of the aqueous extract of the bark of TA. From the plot, it is evident that with the increase in concentration of the aqueous TA extract the inhibition of DPPH radical increases. Another known free radical scavenger, vitamin C was also used in the study. The figure also shows that the extract has comparable radical scavenging activity like vitamin C.

### Dose-dependent preventive activity of the aqueous TA extract

**Figure 2:**
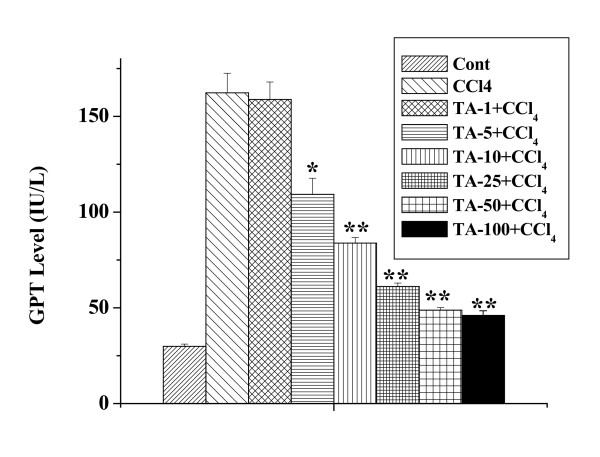
Dose-dependent effect of aqueous TA extract on GPT level against CCl_4 _induced toxicity. Cont: GPT level in normal mice, CCl_4_: GPT level in CCl_4 _treated mice, TA-1+CCl_4_, TA-5+CCl_4_, TA-10+CCl_4_, TA-25+CCl_4_, TA-50+CCl_4 _and TA-100+CCl_4_: GPT levels in extract treated mice applied for 7 days at a dose of 1, 5, 10, 25, 50 and 100 mg/kg body weight respectively before CCl_4 _administration (1 ml/kg body weight). Each column represents mean ± SD, n = 6; (P* < 0.01, P** < 0.001).

Figure 2 shows the dose dependent preventive effect of the extract against CCl_4 _intoxication. To determine at which dose the extract exerts its maximum protective activity, different doses were administered to six different groups of mice. The extract was administered (1, 5, 10, 25, 50, and 100 mg/kg body weight) for 7 days before CCl_4 _(1 ml/kg body weight) intoxication. The preventive effect was found to be the most when the extract was administered at a dose of 50 mg/kg body weight prior to toxin administration. The GPT values in figure 2 confirm the results.

### Time-dependent preventive activity of the aqueous TA extract

**Figure 3:**
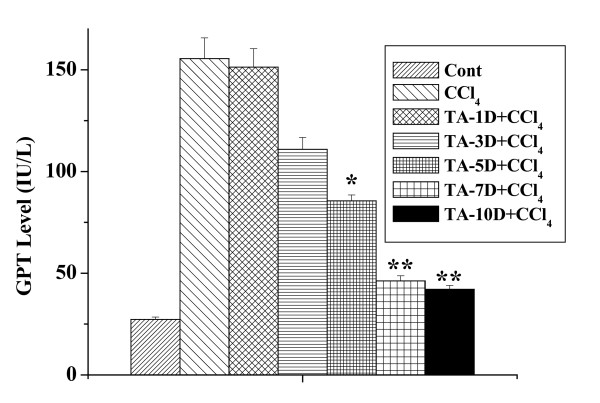
Time-dependent effect of aqueous TA extract on GPT level against CCl_4 _induced toxicity. Cont: GPT level in normal mice, CCl_4_: GPT level in CCl_4 _treated mice, TA-1D+CCl_4_, TA-3D+CCl_4,_TA-5D+CCl_4_, TA-7D+CCl_4 _and TA-10D+CCl_4_: GPT levels in extract treated mice applied for 1, 3, 5, 7 and 10 days respectively at a dose of 50 mg/kg body weight before CCl_4 _(1 ml/kg body weight) administration. Each column represents mean ± SD, n = 6; (P* < 0.01, P** < 0.001).

Figure 3 shows the time-dependent preventive role of the extract against CCl_4 _intoxication. The extract was administered at a dose of 50 mg/kg body weight for 1, 3, 5, 7 and 10 days prior to CCl_4 _(1 ml/kg body weight) intoxication. Administration of the extract prior to CCl_4 _intoxication significantly prevented the disorder caused by the toxin as evident from the lower levels of marker enzyme, GPT in the blood serum. The maximum preventive effect was observed when the extract was given for 7 days before toxin administration.

### Effect of the aqueous TA extract on ALP level

**Figure 4:**
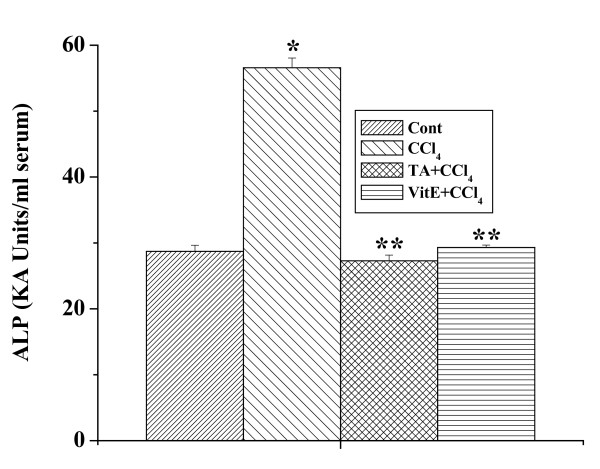
Effect of aqueous TA extract on ALP activity in blood serum and against CCl_4 _intoxication (1 ml/kg body weight). Cont: ALP level in normal mice, CCl_4_: ALP level in CCl_4 _treated mice, TA+CCl_4_: ALP level in which extract was given at a dose 50 mg/kg body weight prior to CCl_4 _administration, VitE+CCl_4_: ALP level in which vitamin E was given at a dose of 200 mg/kg body weight prior to CCl_4 _administration. Each column represents mean ± SD, n = 6; (P* < 0.01, P** < 0.001).

The ALP levels in all the experimental mice are shown in figure 4. In CCl_4 _treated group, elevation of ALP level was observed compared to the normal control group, whereas ALP level decreased in the group of mice pretreated with the aqueous extract for 7 days followed by CCl_4 _administration of 2 days. Pretreatment with vitamin E also reduced the elevated level of ALP.

### Effect of the aqueous TA extract on SOD activity

**Figure 5:**
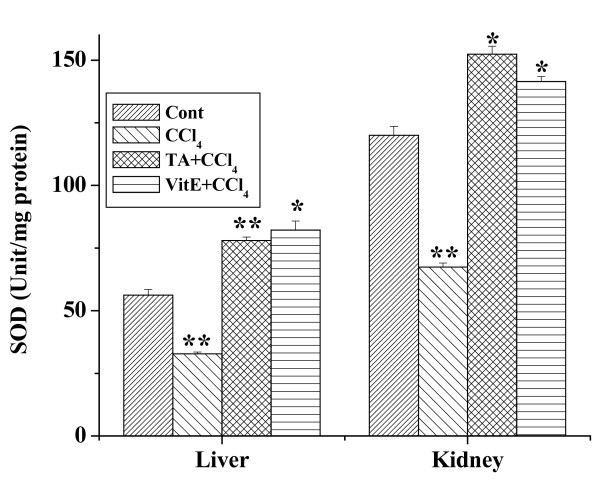
Effect of aqueous TA extract on the SOD levels against CCl_4 _induced hepatic and renal damages in mice. Aqueous extract of TA was administered orally 7 days prior to CCl_4 _treatment (1 ml/kg body weight). For experimental detail, see the materials and methods. Left panel shows the effects of the extract on liver and right panel shows that on the kidney against CCl_4 _induced SOD levels. Cont: SOD level in normal mice, CCl_4_: SOD level in only CCl_4 _treated mice, TA+CCl_4_: SOD level in which extract was given at a dose 50 mg/kg body weight prior to CCl_4 _administration, VitE+CCl_4_: SOD level in which vitamin E was given at a dose of 200 mg/kg body weight prior to CCl_4 _administration. Each column represents mean ± SD, n = 6; (P* < 0.01, P** < 0.001).

The activities of SOD in the tissue homogenates of all experimental mice are shown in figure 5. In the liver homogenate CCl_4 _treatment caused reduction of the SOD activity compared to the normal controls. Enhancement of SOD activity was observed in case of 7 days treatment of aqueous extract prior to the CCl_4 _administration. In the kidney tissue homogenate the SOD activity in the CCl_4 _treated mice was reduced compared to the normal. Mice receiving the aqueous extract prior to CCl_4 _showed an increase in SOD value compared to the CCl_4 _treated mice. Similar results were obtained with the antioxidant vitamin E.

### Effect of the aqueous TA extract on CAT activity

**Figure 6:**
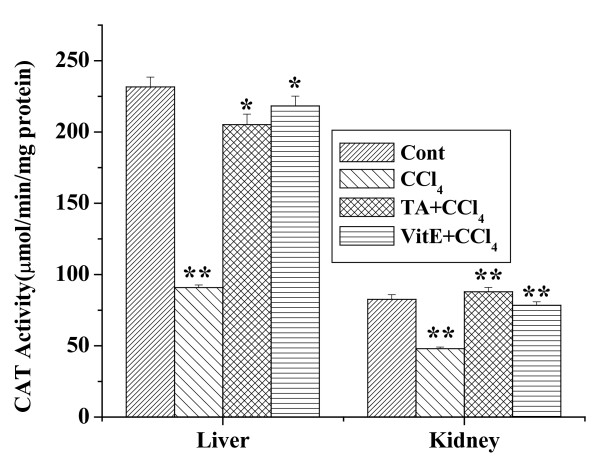
Effect of aqueous TA extract on the CAT levels in CCl_4 _induced hepatic and renal damages in mice. Aqueous extract of TA was administered orally 7 days prior to CCl_4 _treatment (1 ml/kg body weight). For experimental detail, see the materials and methods. Left panel shows the effects of the extract on liver and right panel shows that on the kidney against CCl_4 _induced CAT levels. Cont: CAT level in normal mice, CCl_4_: CAT level in only CCl_4 _treated mice, TA+CCl_4_: CAT level in which extract was given at a dose 50 mg/kg body weight prior to CCl_4 _administration, VitE+CCl_4_: CAT level in which vitamin E was given at a dose of 200 mg/kg body weight prior to CCl_4 _administration. Each column represents mean ± SD, n = 6; (P* < 0.01, P** < 0.001).

CAT activities in the liver and kidney homogenates of mice for all experimental groups are shown in the figure 6. The CAT activity in the liver tissue homogenates of CCl_4 _treated mice was considerably lower than that of normal control mice. In the pretreated group, which got the aqueous extract for 7 days prior to CCl_4_, CAT activity was significantly higher compared to the CCl_4 _treated group. The CAT activity in the kidney homogenates of CCl_4 _treated mice was also lower than the normal control group. The aqueous extract pretreated group showed increased CAT activity, compared to the CCl_4 _treated group and it was almost close to normal group. Vitamin E treatment prior to CCl_4 _intoxication prevented the change in CAT activity in either the liver and or the kidney homogenates.

### Effect of the aqueous TA extract on GST activity

**Figure 7:**
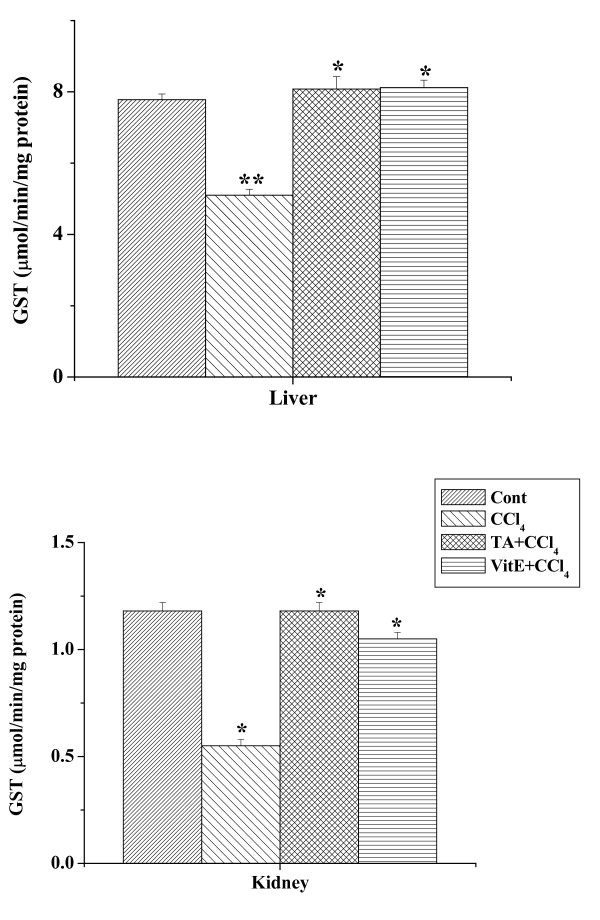
Effect of aqueous TA extract on the GST levels in CCl_4 _induced hepatic and renal damages in mice. Aqueous extract of TA was administered orally 7 days prior to CCl_4 _treatment (1 ml/kg body weight). For experimental detail, see the materials and methods. Left panel shows the effects of the extract on liver and right panel shows that on the kidney against CCl_4 _induced GST levels. Cont: GST level in normal mice, CCl_4_: GST level in only CCl_4 _treated mice, TA+CCl_4_: GST level in which extract was given at a dose 50 mg/kg body weight prior to CCl_4 _administration, VitE+CCl_4_: GST level in which vitamin E was given at a dose of 200 mg/kg body weight prior to CCl_4 _administration. Each column represents mean ± SD, n = 6; (P* < 0.01, P** < 0.001).

GST activity as measured from the liver and kidney tissue homogenates of all the experimental mice have been shown in figure 7. In the liver homogenates decreased GST activity was observed in CCl_4 _treated mice compared to the normal control group. Pretreatment with the aqueous extract for 7 days prior to CCl_4 _intoxication enhanced that activity significantly. In the kidney homogenates GST activity of CCl_4 _treated group was lower compared to that in the normal group, while the GST activity was found to be increased in the kidney homogenate of mice treated with aqueous extract for 7 days prior to CCl_4 _treatment. GST activity in vitamin E pretreated group was close to the normal level.

### Effect of the aqueous TA extract on GSH level

**Figure 8:**
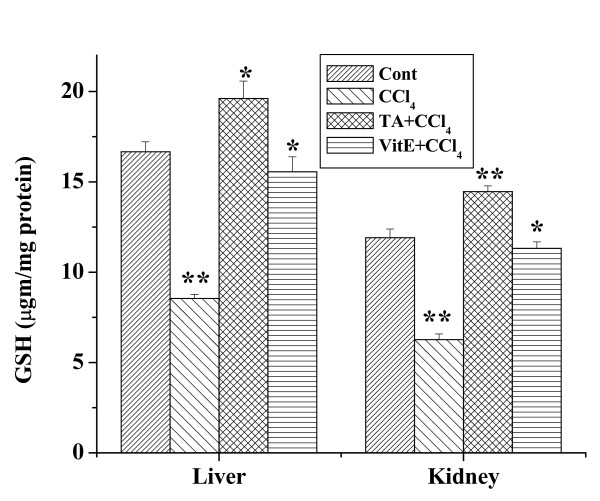
Effect of aqueous TA extract on the GSH levels in CCl_4 _induced hepatic and renal damages in mice. Aqueous extract of TA was administered orally 7 days prior to CCl_4 _treatment (1 ml/kg body weight). For experimental detail, see the materials and methods. Left panel shows the effects of the extract on liver and right panel shows that on the kidney against CCl_4 _induced GSH levels. Cont: GSH level in normal mice, CCl_4_: GSH level in only CCl_4 _treated mice, TA+CCl_4_: GSH level in which extract was given at a dose 50 mg/kg body weight prior to CCl_4 _administration, VitE+CCl_4_: GSH level in which vitamin E was given at a dose of 200 mg/kg body weight to CCl_4 _administration. Each column represents mean ± SD, n = 6; (P* < 0.01, P** < 0.001).

Effects of the aqueous extract on GSH level for all experimental groups are shown in figure 8. CCl_4 _treatment caused significant decrease of GSH level in liver tissue homogenates compared to the normal group. Pretreatment of aqueous extract for 7 days followed by 2 days CCl_4 _treatment enhanced the level of GSH compared to CCl_4 _treated group. Like liver, the GSH level in CCl_4 _treated group was also lower than the normal. Treatment of the aqueous extract prior to the CCl_4_treatment increased the GSH level. Similar results were obtained with vitamin E.

### Effect of the aqueous TA extract on lipid peroxidation

**Figure 9:**
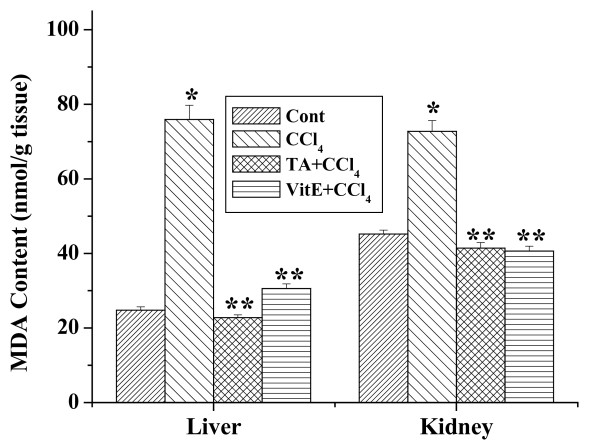
Effect of aqueous TA extract on TBARS formation in CCl_4 _induced hepatic and renal damages in mice. Aqueous extract of TAwas administered 7 days prior to CCl_4 _treatment (1 ml/kg body weight). Left panel shows effects of the extract on liver and right panel shows that on the kidney against CCl_4 _induced MDA contents. Cont: MDA content in normal mice, CCl_4_: MDA content in only CCl_4 _treated mice, TA+CCl_4_: MDA level in which extract was given at a dose 50 mg/kg body weight prior to CCl_4 _administration, VitE+CCl_4_: MDA level in which vitamin E was given at a dose of 200 mg/kg body weight prior to CCl_4 _administration. Each column represents mean ± SD, n = 6; (P* < 0.01, P** < 0.001).

TBARS concentrations (expressed as MDA) in the liver and kidney homogenates of all experimental mice are shown in figure 9. In liver tissue homogenates of CCl_4 _treated group elevation of MDA level was observed compared to the normal control. Pretreatment of aqueous extract for 7 days followed by CCl_4 _treatment for 2 days decreased the level of MDA compared to CCl_4 _treated group. Likewise, in the kidney tissue homogenates the MDA level in CCl_4 _treated group was also higher than the normal group. Treatment of the aqueous extract prior to the CCl_4 _administration decreased the enhanced MDA level significantly. Similar results were obtained with vitamin E.

## Discussion

Present study was conducted to evaluate the protective effect of the aqueous extract of TA against CCl_4 _induced hepatic and renal disorders in mice. Results suggest that the extract possesses protective action against both hepatic and renal dysfunctions induced by the potent toxin, CCl_4_. Data showed that the extract responds in both dose- and time-dependent manner. Maximum protective activity of the extract was obtained when administered once daily at a dose of 50 mg/kg body weight for 7 days before toxin administration as revealed from the figures 2 and 3. This dose and time have been followed for the subsequent studies.

A number of chemicals including various environmental toxicants and clinically useful drugs can cause severe cellular damages in different organs of our body through the metabolic activation to highly reactive substances such as free radicals. CCl_4 _is one of such extensively studied environmental toxicant. The reactive metabolite trichloromethyl radical (^.^CCl_3_) has been formed from the metabolic conversion of CCl_4 _by cytochrome P-450 [50]. As O_2 _tension rises, a greater fraction of ^.^CCl_3 _present in the system reacts very rapidly with O_2 _and many orders of magnitude more reactive free radical, CCl_3_OO^. ^has been generated from ^.^CCl_3 _[51]. These free radicals initiate the peroxidation of membrane poly-unsaturated fatty acids (PUFA) [52], generates PUFA^. ^and covalently bind to microsomal lipids and proteins [53]. This phenomenon results in the generation of ROS, (like the superoxide anion O_2_^-^, H_2_O_2 _and the hydroxyl radical, ^.^OH). Evidence suggests that various enzymatic and non-enzymatic systems have been developed by the cell to cope up with the ROS and other free radicals. However, when a condition of oxidative stress establishes, the defense capacities against ROS becomes insufficient [54]. ROS also affects the antioxidant defense mechanisms, reduces the intracellular concentration of GSH and decreases the activity of SOD and CAT. It has also been known to decrease the detoxification system produced by GST [55]. Increasing evidence indicates that oxidative stress causes organ injury and carcinogenesis [56].

In the present study, it has been observed that CCl_4 _induced a significant elevation of the levels of serum marker enzymes, GPT and ALP. In addition, this potent toxicant caused significant decrease in SOD, CAT and GST activities, depleted the GSH content and enhanced lipid peroxidation in both liver and kidney. We also determined the levels of the markers related to renal damages. However, under the present experimental conditions (1 ml/kg body weight for 2 days), there was no change in either the urea nitrogen or creatinine (non protein nitrogen) in the serum of CCl_4_intoxicated animals. Besides, we did not find any change in the creatinine level in the urine of the experimental animals (data not shown) as well. The exact reason for this is not clearly known. One possibility is that, the time of CCl_4 _exposure to the animals was not enough for the renal damage although oxidative stress could be induced by that exposure. Tirkey et al [57] have recently conducted experiments to determine the effect of CCl_4 _on the renal damages in rats and obtained similar results.

It has been reported that SOD, CAT and GST constitute a mutually supportive team of defense against ROS [58,59]. The decreased activity of SOD in liver and kidney in CCl_4 _treated mice may be due to the enhanced lipid peroxidation or inactivation of the antioxidative enzymes. This would cause an increased accumulation of superoxide radicals, which could further stimulate lipid peroxidation. GST binds to liophilic compounds and acts as an enzyme for GSH conjugation reactions [60]. Decrease in GSH activity during CCl_4 _toxicity might be due to the decreased availability of GSH resulted during the enhanced lipid peroxidation. Administration of the aqueous extract of TA prior to CCl_4 _intoxication could not only prevent the CCl_4 _induced increased levels of serum marker enzymes GPT and ALP, but also protected the antioxidant machineries of the liver and kidney as revealed from the enhanced levels of SOD, CAT and GST activities, increased level of GSH content and decreased level of lipid peroxidation. Besides, the extract showed radical scavenging activity by reacting with DPPH and this scavenging activity is comparable to that of a potent free radical scavenger, vitamin C in cell free system.

In the liver, CCl_4 _is metabolized by the cytochrome P450-dependent monooxygenase systems followed by its conversion to more chemically active form, trichloromethyl radical (^.^CCl_3_) [61]. The enzymes involved in this process are located in the endoplasmic reticulum of the liver and their activities are dependent on many environmental factors. Some herbal extracts are known to prevent the oxidative damages in different organs by altering the levels of cytochrome P-450 through their antioxidant properties [20]. Our results suggest that the aqueous extract of the bark of TA possesses potent antioxidative activity and protects liver and kidney against CCl_4 _induced oxidative stress probably via the alteration of cytochrome P-450.

## Conclusion

Combining all, we would like to say that the aqueous extract of TA protects liver and kidney tissues against oxidative damages and could be used as an effective protector against CCl_4 _induced hepatic and renal damages. Further works are needed to fully characterize the responsible active principle(s) present in the plant and elucidate its possible mode of action and that is in progress.

## Competing interests

The author(s) declare that they have no competing interests.

## Authors' contributions

PM: made substantial contributions to the design of the study, the collection of the data as well as the interpretation and analysis of the data. He also drafted the manuscript and gave final approval for its submission to the Journal for consideration of publication.

MS: made substantial contributions to the design of the study, the collection of the data as well as the interpretation and analysis of the data. She also drafted the manuscript and gave final approval for its submission to the Journal for consideration of publication.

PCS: the investigation-in-charge for the study, made substantial contributions to the design of the study, as well as the interpretation and analysis of the data. He also drafted the manuscript and gave final approval for its submission to the Journal for consideration of publication.

All authors read and approved the final manuscript.

## Pre-publication history

The pre-publication history for this paper can be accessed here:


